# A Microchip for Integrated Single-Cell Gene Expression Profiling and Genotoxicity Detection

**DOI:** 10.3390/s16091489

**Published:** 2016-09-14

**Authors:** Hui Dong, Hao Sun

**Affiliations:** School of Mechanical Engineering and Automation, Fuzhou University, Fujian 350116, China; hdong@fzu.edu.cn

**Keywords:** single-cell analysis, integrated multiplex RT-qPCR, microfluidics

## Abstract

Microfluidics-based single-cell study is an emerging approach in personalized treatment or precision medicine studies. Single-cell gene expression holds a potential to provide treatment selections with maximized efficacy to help cancer patients based on a genetic understanding of their disease. This work presents a multi-layer microchip for single-cell multiplexed gene expression profiling and genotoxicity detection. Treated by three drug reagents (i.e., methyl methanesulfonate, docetaxel and colchicine) with varied concentrations and time lengths, individual human cancer cells (MDA-MB-231) are lysed on-chip, and the released mRNA templates are captured and reversely transcribed into single strand DNA. Glyceraldehyde-3-phosphate dehydrogenase (*GAPDH*), cyclin-dependent kinase inhibitor 1A (*CDKN1A*), and aurora kinase A (*AURKA*) genes from single cells are amplified and real-time quantified through multiplex polymerase chain reaction. The microchip is capable of integrating all steps of single-cell multiplexed gene expression profiling, and providing precision detection of drug induced genotoxic stress. Throughput has been set to be 18, and can be further increased following the same approach. Numerical simulation of on-chip single cell trapping and heat transfer has been employed to evaluate the chip design and operation.

## 1. Introduction

As a leading cause of death worldwide, cancer has been widely recognized as a disease from patient-specific mutations [1]. The transformation from normal cells into tumor cells is the result of the interaction between personal genetic factors and external carcinogens [2]. Traditionally, cell population or bulk tissue samples were used to investigate genetic variations and evaluate prognosis in laboratories or clinical settings providing an averaged signal from a cell group [3,4] While cells may appear morphologically identical, recent evidence reveals that genetic activity of individual cells in a population can vary due to sample heterogeneity or clonal diversity [5,6]. Gene expression measurement using groups of cells failed to reveal mechanisms by which heterogeneity affects the therapeutic outcome [7]. Fueled by cutting-edge cellular and molecular technologies, the data of single cancer cells has transformed from qualitative microscopic readouts to quantitative genomic datasets. Particularly, single-cell gene expression profiling promises to address the issues in cancer research, including unveiling intra-tumor heterogeneity [8], tracing cell lineages [9], interpreting rare tumor cell populations [10], and quantifying mutation rates [11]. However, such assays have been technically challenging due to the low quantity and degradation of messenger RNA (mRNA) from an individual cell [12]. A typical mammalian cell contains about 10–30 pg RNA and mRNA accounts for 1%–5% of the total cellular RNA depending on the cell type and physiological state [13].

Microfluidics or lab-on-a-chip technology, which is characterized by systems with dimensions of 500 μm to a few millimetres [14], presents a unique and powerful platform for working with living cells. To date, microfluidics has been successfully employed to detect complex biological processes in human cancers [15]. Particularly, microchips for single-cell isolation [16,17], RNA sequencing [18,19], DNA detection [20,21], and protein analysis [22,23] have been fabricated and tested. For cell isolation, immunocapture [24,25], and label-free-based methods (identify cells by size, electrical polarizability, and hydrodynamic properties) [26,27] have been incorporated in microfluidics for single cell isolation. Generally, the immunocapture approach requires additional sample preparation steps and may potentially compromise cell viability for accurate on-chip single-cell processing. Recently, there has been interest in cell trapping methods which avoid the use of biochemical labels^26^. For on-chip single-cell gene expression profiling, the main limitations in current microfluidic based single-cell study are integration [20,28] and testing throughput [29]. In parallel, commercial systems have been created for microfluidic single-cell gene expression profiling [30]. Particularly, the nCounter^®^ (NanoString Technology Inc., Seattle, WA, USA) system has been developed by NanoString Inc. for direct quantitation of target transcripts. Although up to 800 genes from total RNA, cell lysate, or whole-blood lysate can be detected simultaneously, the recommended amount of starting material is 100 ng purified total RNA, or lysate from 10,000 cells (NanoString Technology Inc., Seattle, WA, USA) with an assay volume of 30 μL limiting the application in single-cell processing and analysis. Currently, for gene expression at the single-cell level, the most advanced commercial tools are C1^TM^ and BioMark HD^TM^ developed by Fluidigm company (South San Francisco, CA, USA). By combining IFCs (integrated fluidic circuits) with commercial systems, targeted genes can be amplified and real-time quantified. While IFCs are well designed and operated, the testing throughput and experimental cost per test are difficult to be custom-tailored. Additionally, for a whole RT-qPCR sample transfer of preamplification was requested [31], indicating the test integrality can be further improved. Thus, there is still a need for developing simple and economic methods for daily tests in laboratories.

In our previous work, we proposed microchips for single-cell gene expression profiling [32,33,34]. Here, we present a microchip for multiplexed single-cell analysis. Following cell immobilization, single human cancer cells were treated by methyl methanesulfonate (MMS), docetaxel (TXT), and colchicine (COL), with different doses and time lengths. Then, in the same chip, six treated individual cells were lysed and mRNA templates in cell lysate were captured by a solid-phase method. Consequently, reverse transcription and multiplex PCR were performed in a single chamber. The modification in expression levels of glyceraldehyde 3-phosphate dehydrogenase (*GAPDH*), cyclin-dependent kinase inhibitor 1A (*CDKN1A*), and serine/threonine-protein kinase aurora-A (*AURKA*) by drug treatment have been studied. The microchip can integrate all steps of single-cell gene expression analysis with a tripled throughput (18 readouts per run) by employing identical time lengths and amounts of micro-chambers reported previously. To evaluate the chip design and operation, fluid-structure interaction of on-chip single cells with surrounding microflow has been studied by a fluid-structure interaction simulation.

## 2. Principle, Materials, and Experimental Setup

### 2.1. Principle

The cultured individual human cancer cells were isolated and immobilized on the microchip. Then, the cells were chemically lysed and the released mRNA templates were captured and purified by magnetic microbeads. In a same reaction zones, reverse transcription (RT) and multiplex qPCR were performed in sequence. During the RT process, mRNA templates from cell lysis were bound to the surface of the beads via the oligo dT primers. During multiplex qPCR, three targeted genes (*GAPDH, CDKN1A, AURKA*) were amplified in a single reaction chamber. The amplification of three targets were real-time detected using sequence-specific probe/primer sets. By the selected filters, signals from three reporter dyes (FAM, HEX, CY3) can be acquired and the grey values of images were measured in Image-J software (National Institutes of Health, Bethesda, MD, USA). To correct for fluorescent fluctuations due to batch-to-batch changes in cavity volume and PCR component concentrations, a passive reference dye (ROX) was employed to normalize the reporter signal during real-time measurements. 

**Figure 1:**
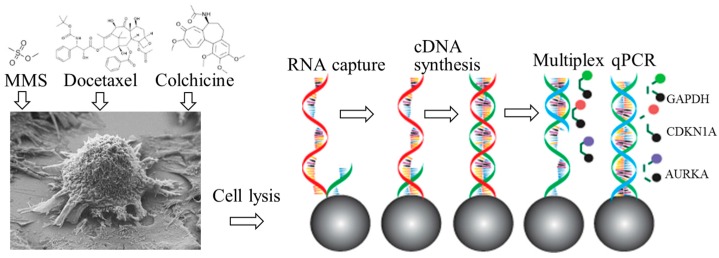
Principle of microfluidic integrated single-cell gene expression profiling. The human cancer cells were isolated and immobilized in the microchip. Then, the cells were chemically lysed and the released mRNA templates were captured and purified by magnetic microbeads followed by RT and multiplex qPCR. The amplification of three targets (*GAPDH, CDKN1A, AURKA*) were detected in real-time using sequence-specific probe/primer sets.

Furthermore, based on the hypothesis that gene expression profiles can be used to interpret mechanisms of genotoxicity [35,36], drug treatments were employed to study the dose-dependent and time-dependent patterns of gene expression. By treating human cancer cells with three drugs (MMS, TXT, and COL) at varied concentrations for different time lengths, *GAPDH, CDKN1A*, and *AURKA* genes were leveraged for assessing the genotoxicity at the single-cell level. All steps of single-cell isolation, immobilization and lysis, mRNA capture, cDNA synthesis, target gene amplification, and detection were fully integrated on the microchip. The cultured cells, the working principle, and the corresponding experimental setup are demonstrated in Figure 1.

### 2.2. Materials and Experimental Set-up

The MDA-MB-231 cell line was obtained from the American Type Culture Collection^®^ (Manassas, VA, USA). Leibovitz's L-15 Medium, fetal bovine serum (FBS), penicillin-streptomycin (P/S, penicillin 10^4^ unit/mL, streptomycin 10^4^ mg/mL), 0.25% trypsin-ethylenediaminetetraacetic acid (EDTA), Dulbecco’s phosphate-buffered saline (D-PBS), cell lysis buffer, Vybrant multicolor cell-labeling kit (DiI, DiO and DiD), Dynabeads^®^ (61005) mRNA Kit, TaqMan^®^ reverse transcription reagents (4304134), XenoRNA control (10^5^ copies/µL), TaqMan^®^ Gene Expression master mix (4369016), thin-walled RNase-free PCR Tubes (0.2 mL), RNase-free water, RNaseZap Wipes, and MicroAmp^®^ optical adhesive film (4311971) for the evaporation barrier were purchased from Thermo Fisher Scientific Inc. (Grand Island, NY, USA). The target-specific primer sets of *GAPDH* (forward: aatcccatcaccatcttccag, reverse: aaatgagccccagccttc), *CDKN1A* (forward: cccttgtcctttccctt cag, reverse: cttgccctgaggttag aactag) and *AURKA* (forward: gtacatgctccatcttc cagg, reverse: aaagaactccaaggctccag) were designed on-line by the RealTime PCR Tool from Integrated DNA Technologies (IDT, Coralville, IA, USA). Additionally, the probe sets of *GAPDH* (ccagcatcgccccacttgatttt, HEX/BHQ-2), *CDKN1A* (ttccccttcccagtccattgagc, FAM/BHQ-1) and *AURKA* (caccttctcatc atgcatccgacctt, CY3/BHQ-2) were acquired by the IDT software, and synthesized by SBS Genetech Co., Ltd (Beijing, China). MMS (99%) and bovine serum albumin (BSA, 98%, V900933) were obtained from Sigma-Aldrich (St. Louis, MO, USA). Docetaxel (2 mg, 98%) and colchicine (2 mg 95%) were ordered from Topscience Co., Ltd. (Shanghai, China). Polydimethylsiloxane (PDMS, SYLGARD184) was purchased from Dow Corning Corporation (Auburn, MI, USA). SU-8 photoepoxy GM 1075 (1000 mL) was from Gersteltec Sarl (Pully, Switzerland). AZ4620 (500 mL) and Shipley S1805 (1000 mL) were from MicroChemicals Inc. (Ulm, Germany).

**Figure 2:**
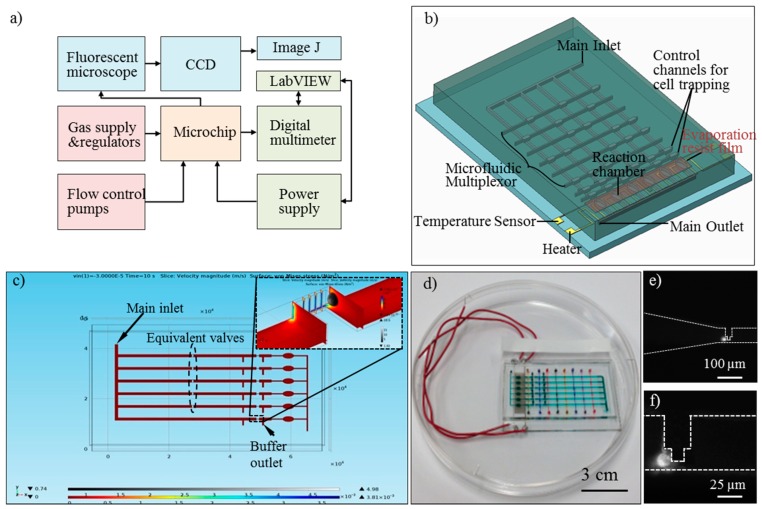
(**a**) Schematic of the experimental setup (arrow symbol means ‘input’); (**b**) Schematic of the multilayer microchip; (**c**) 3-D transient simulation results of on-chip single cell trapping in COMSOL software; (**d**) A fabricated microchip prototype; (**e**) An on-chip immobilized single cell; and (**f**) Details of the trapping zone and the single cell.

Closed-loop temperature control of the device chambers was achieved using the integrated temperature sensor and heater with a proportional-integral-derivative (PID) algorithm implemented in a LabVIEW (National Instruments Corp., Austin, TX, USA) program on a personal computer. The resistance of the sensor was measured by a digital multimeter (34420A, Agilent Technologies Inc., Santa Clara, CA, USA), and the heater was connected to a DC power supply (E3631, Agilent Technologies). The microfluidic valves of the device were controlled by individual gas pressure regulators (Concoa, Virginia Beach, VA, USA) interfaced via 20 gauge stainless steel tubing (Becton Dickinson, Franklin Lakes, NJ, USA) and Tygon tubing (ID: 0.79 mm, OD: 2.38 mm, Saint-Gobain, Grand Island, NY, USA). The inlets and outlets of the device were sealed off by polycarbonate plugs (diameter: 1 mm). The fluorescent intensity of the reaction was measured from images acquired by an inverted epifluorescence microscope (IX81, Olympus, Center Valley, PA, USA) with a CCD camera (c8484, Hamamatsu, Boston, MA, USA) of the reaction chamber. The schematic of the experimental setup is shown in Figure 2a.

## 3. Chip Design, Simulation, and Fabrication

### 3.1. Design

In L-edit software (Tanner Research, Inc., Monrovia, CA, USA), a multi-layer microchip with six testing units and one inlet and outlet pair was designed (Section 1 in Supplementary Materials). The architecture of the multi-layer microchip is shown in Figure 2b. SiO_2_ substrate, serpentine-shape Au/Cr microheater and temperature sensor, SU-8 passivation thin film, flow layer, evaporation resist film, and pneumatic control layer were packaged from the bottom, up. Within each testing unit of the flow layer, a cell processing component (for cell isolation and immobilization) and a ‘vesica piscis’ shaped reaction chamber (265/153 in length/width) were connected by microchannels, which can be partitioned by pneumatically controlled valves (1 × 0.7 mm). In the ceiling of the reaction components, an evaporation resist film (4 × 0.5 cm) was embedded, which serves as a barrier to minimize evaporation and associated reagent loss during thermal cycling. In the control layer, eight individually pressurized elastomeric binary valves are arranged in a combinatorial array. The hole diameter of inlets and outlets were designed to be 0.8 mm identically. Compared with existing commercial chips (IFCs, Fluidigm), the design employed only one chamber and five valves for each working unit, which is more simplified than IFCs’ (six chambers and seven valves). Therefore, the design can assemble more channels and units on an identical substrate, and holds a potential for accessing higher throughputs of qPCR tests. The microfluidic operating procedures of the chip are similar to previous work.

### 3.2. Cell Trapping Simulation

Theoretical analysis of single-cell trapping process can be used to verify microchip design, optimize microfluidic control strategy, and evaluate cell viability. Once introduced to the chip, the interaction (fluid-structure interaction, FSI) of the deformable cell with the surrounding fluid flow occurs. Fluid flow causes deformation of the structure which, in turn, changes the fluid flow boundary conditions (BCs). As FSI problems in general are often too complex to solve analytically, numerical simulation has been employed here. This FSI coupling appears on the interface of fluid and cell, and the fluid defines the load on the solid surface while the displacement and velocity of solid is transmitted to fluid. The theoretical fundamentals are included in Section 2 in Supplementary Materials. In Comsol Multiphysics software (Burlington, MA, USA), a 3-D Finite Element model was setup and solved by the transient method. With an inlet flow velocity at 3 × 10^−5^ m/s, the magnitude of flow velocity and von Mises stress on cell surface at 10 second were presented in Figure 2c. The results verified that with an inlet velocity below 12 × 10^−5^ m/s, the equivalent von Mises stress was 19.8 dyn/cm^2^, which was found to be around the stress acting on normal human vascular endothelial cells [37]. For inlet flow with higher velocity, the stress would impair cell viability [38].

### 3.3. Device Fabrication

Microchip fabrication followed the standard multi-layer soft lithography techniques [39]. Chrome (20 nm) and gold (110 nm) thin films were deposited and patterned onto a glass slide (Fisher HealthCare, Houston, TX, USA) followed by SU-8/PDMS passivation. AZ 4620 photoresist was first spin-coated and patterned. Once developed, the photoresist was heated up to 200 °C for 1 h at which temperature the reflowing of the photoresist formed flow channels with a rounded cross-section. Then, on the same wafer, SU-8 photoresist was spin-coated and patterned to define the other parts of the flow channel. Then, PDMS was poured over the molds twice and an additional vapor barrier was embedded in the flow layer PDMS. Sheets bearing the microfluidic features were then peeled off the mold followed by inlet and outlet hole punching. Additionally, uncured PDMS was spun on a wafer to form a featureless membrane (20 μm in thickness). The membrane was then sandwiched between the flow and control layer by oxygen plasma. Finally, the PDMS device was bonded to the heater and sensor by oxygen plasma resulting in a packaged device. The details of the fabrication process are included in Section 3 in Supplementary Materials, and a microchip prototype is shown in Figure 2d.

## 4. Results and Discussion

### 4.1. Single Cell Trapping Test

**Figure 3:**
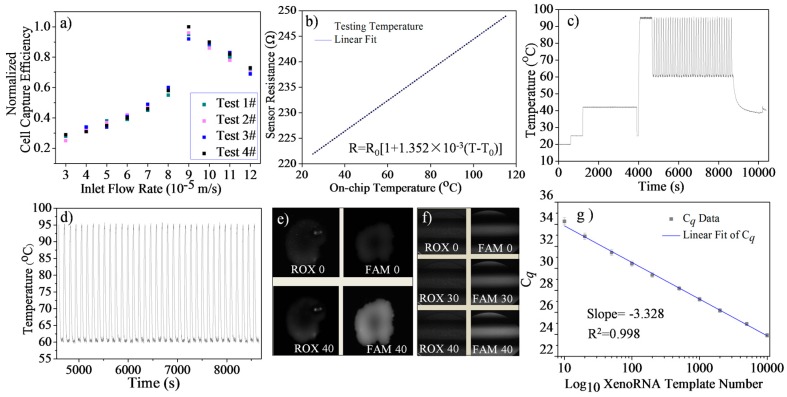
(**a**) Cell trapping efficiency with different inlet flow rate; (**b**) Temperature sensor calibration; (**c**) On-chip temperature control evaluation; (**d**) Detailed temperature history of a 35-cycle on-chip PCR; (**e**) On-chip RT-PCR validation; (**f**) On-chip RT-qPCR validation (‘0, 30, 40’ indicates cycle number); and (**g**) Standard curve of on-chip XenoRNA amplification.

Cell suspension (10^6^ cells/mL) was treated with Vybrant dye at a volume ratio of 1:200. Then, with different carrier flow velocities corresponding to the simulation results (3~12 × 10^−5^ m/s), cells were dispensed at a fixed cell density (10^4^ cells/mL) and transported to the trapping region. Repeated experiments were conducted, in each of which a dilute cell suspension was introduced into the device for cell trapping (Figure 2e,f). The ratio of the number of experiments in which a single cell was successfully trapped to the total number of experiments provided a measure of cell trapping probability. Higher flow rates were found to cause a lower trapping probability as cells tended to pass through the trap because of the increased cell deformation caused by the flow. However, a lower flow rate would require a longer trapping time. Practically, in this work, the total cell trapping time ranged from ~1 min to tens of minutes with the inlet flow velocity changed from 30 to 120 μm/s. Undesirable fluctuations in the microenvironmental temperature and CO_2_ concentration can impair cell viability (affecting gene expression results eventually) when cells are exposed outside the incubator [40,41] and, thus, it is necessary to optimize the cell trapping process. Here, we defined a normalized trapping efficiency by *ε = (ρ*/*ρ_max_)*/*(t/t_max_)*, where *ρ* is the trapping probability, and *t* is the trapping time. This parameter was found to increase with the flow rate until reaching the 100% maximum at 90 µm/s, and then decreased as the inlet flow rate further increased (Figure 3a). The optimum flow rate of 90 µm/s for cell suspensions of 10^4^ cells/mL in concentration was used in all subsequent single-cell gene expression analysis experiments. 

### 4.2. Temperature Sensor Calibration

The chip was fixed in a temperature-controlled environmental chamber (Delta 9023, Delta Design Inc., Poway, CA, USA). Using platinum resistance temperature detector probes, the temperature of the chamber and the corresponding on-chip resistance was determined by digital multimeter. The measured resistance (R) of the gold temperature sensor was observed to vary linearly with temperature (T). The dependence could be represented by *R = R*_0_ [*1* + *α* (*T* − *T*_0_)], where *R*_0_ (221.9 Ω) is the sensor resistance at a reference temperature *T*_0_ (25.1 °C), and *α* (1.352 × 10^−3^ 1/°C) is the temperature coefficient of resistance of the sensor. Fitting this relationship to the measurement data determined the values of the parameters, which were used to determine the chamber temperature from the measured sensor resistance during single-cell RT-qPCR experiments. The temperature sensor calibration curve is shown in Figure 3b. 

### 4.3. Temperature Control Evaluation

In LabVIEW software, a graphical program designed for automatic on-chip temperature control is presented in Section 4 in Supplementary Materials. The accuracy and precision of the on-chip heating system over the course of RT and 35 consecutive cycles PCR were evaluated based on Figure 3c,d. The accuracy was defined by the difference between the set point and measured average temperature. The precision was defined as the average of the measured standard deviation of the temperature variation at the set point. For the RT step, with set points of 25 °C and 42 °C, we measured the temperature accuracy as 0.11 °C and 0.16 °C, and the precision was 0.08 °C and 0.1 °C, respectively. For the PCR step, the accuracy of the two set points (denaturing at 95 °C, annealing/ extension at 60 °C) was 0.53 and 0.21, and the precision was 0.16 °C and 0.14 °C, respectively. The chip achieved target temperatures with minimal overshoot (<10 s). All of these results indicate that the chamber temperature can be controlled to produce accurate and rapid amplification reactions. 

### 4.4. Microfluidic RT-qPCR Validation using XenoRNA

A XenoRNA hydrolysis primer/probe set and 2 × 10^4^ copies XenoRNA templates were used to validate the feasibility of on-chip RT-PCR. XenoRNA templates were reverse transcribed by RT and amplified via 35 cycles of PCR. The amplification was compared with the NTC (no template control). The fluorescent images and background subtracted fluorescent intensity of the reaction product are shown in Figure 3e and the protocol of the test is shown in Section 5 in Supplementary Materials. For the on-chip RT-PCR of 1 × 10^4^ copies of XenoRNA, the fluorescent image of reporter showed much greater fluorescent intensity (1.71 ± 0.2) than the NTC sample (0 ± 0.05) as shown in Figure S9.

Then, we validated the on-chip RT-qPCR by this approach. In five analysis units of the microchip, we introduced 2 × 10^4^ copies of XenoRNA and 4 × 10^6^ beads. In the sixth analysis unit the same procedures and reagents were used except XenoRNA was not introduced. Fluorescent intensities of reaction chambers were acquired at the end of each PCR cycle. Of these, at the end of cycles 0, 30, and 35, corresponding intensity images from one unit are shown in Figure 3f. All of these results indicate there was a significant amplification of XenoRNA templates and negligible amplification of the NTC in the reaction chamber. 

In addition, we tested on-chip amplification efficiency using XenoRNA templates. Generally, the quantification cycle, C*_q_*, was set as the cycle number at which the measured fluorescence crosses a threshold of 20 σ, where σ is the standard deviation of the fluorescence intensity for the first fifteen PCR cycles [42]. Using dilutions of known XenoRNA templates (10–10,000), we constructed the standard curve by linear fitting of C*_q_* values as shown in Figure 3g. Additionally, PCR efficiency is defined by *E =* (*10 − 1*/*k − 1*) *× 100%*, where *k* is the slope of the C*_q_* as a function of the logarithm of the template copy number. Thus, we determined that the on-chip amplification efficiency of the XenoRNA template was 99.76%. Furthermore, the standard deviation of the C*_q_* value decreased gently from 0.33 to 0.17 with XenoRNA copy number climbing from 10 to 10,000. This phenomenon indicated there was an acceptable higher fluctuation of fluorescence signals in a lower abundance sample testing. In addition, the dynamic range of this on-chip approach was suitable for single-cell genetic analysis as the total amount of a targeted gene within an individual cell was commonly single-digit [43]. More details about the procedures of on-chip RT-qPCR and DNA amplification are discussed in Section 6 in Supplementary Materials.

### 4.5. Repeatability and Reproducibility Test Using XenoRNA

**Figure 4:**
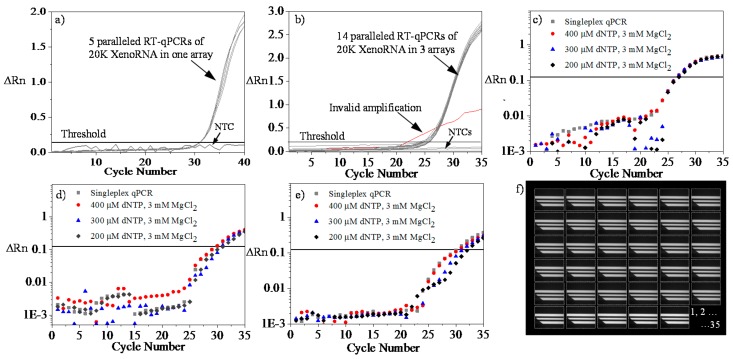
(**a**) Repeatability or intra-assay variation testing of microfluidic RT-qPCR (20,000 copies XenoRNA); (**b**) Reproducibility or inter-assay variation testing of microfluidic RT-qPCR; (**c**) On-chip PCR of *GAPDH* by singleplex or multiplex methods; (**d**) On-chip PCR of *CDKN1A* by singleplex or multiplex methods; (**e**) On-chip PCR of *AURKA* by singleplex or multiplex methods (square points mean the data by singleplex method while the circle, triangle and diamond symbols mean the data by multiplex tests); and (**f**) A fluorescent image set reflecting *CDKN1A* reporter intensity during a 35-cycle qPCR (Numbered as 1, 2, …, 35).

To further investigate the repeatability (i.e., intra-assay variation), we introduced 2 × 10^4^ copies of XenoRNA and 4 × 10^6^ beads to perform parallelized on-chip RT-qPCR. In Figure 4a, the value of ∆Rn, indicating the magnitude of the fluorescent signals and therefore amplification generated by PCR, demonstrates an exponential increase in the amounts of the XenoRNA copies with the cycle number. In this scenario, the threshold value was calculated to be 0.14 and, accordingly, C*_q_* ranged from 25.2 to 25.5 using 2 × 10^4^ copies XenoRNA. The results in Figure 4a demonstrate that with the same amount of homogenized starting templates, the C*_q_* difference for getting significant amplification in all five analysis units was below 0.3. 

Similarly, the reproducibility (inter-assay variation) of the on-chip reaction was studied following identical procedures in three separate chips. The results in Figure 4b demonstrate that with the same amount of homogenized starting templates, the cycle number difference for getting significant amplification in 14 units of three microchips was below 0.4. These results served to verify the proper function of the device for integrated RT-qPCR and to the consistency among the individual units.

### 4.6. Multiplex Amplification

In the reaction chamber, the amplification of the less efficient or less abundant target can be inhibited by the more-efficient or more-abundant target. By comparing the amplification curve of multiplex reaction to the singleplex reaction, the inhibitory effect can be quantified. Thus, to optimize the amplification of *GAPDH*, *CDKN1A*, and *AURKA*, we sequentially increased the concentrations of deoxynucleotides (dNTPs) in PCR master mix while keeping DNA polymerase and MgCl_2_ concentration constant. On-chip 35-cycle qPCR amplification plots of singleplex tests and multiplex tests are shown in Figure 4c–e. For *GAPDH* (Figure 4c), the mean C*_q_* values of singleplex reaction (26.95) and multiplex reaction with different dNTPs concentrations (26.97, 26.98, 26.99 for 200, 300, and 400 µM) were very close, while for *CDKN1A* (Figure 4d), the mean C*_q_* values of singleplex (29.94) was smaller than multiplex reactions with different dNTPs concentrations (31.98, 31.01, 29.96 for 200, 300, and 400 µM). This trend can be also found in *AURKA* testing (Figure 4e), in which singleplex reaction had a C*_q_* value of 30.32, while multiplex had values of 32.28, 31.29, and 30.87 for 200–400µM dNTPs. These results indicate there was a competition of amplification for different targeted genes in the multiple testing. To minimize the impact of dNTP concentration in PCR master mix, we used 400 µM in subsequent on-chip multiplex tests. Typical images of the CDKN1A reporter signal during a 35-cycle multiplex qPCR has been recorded in Figure 4f.

### 4.7. Drug Dose Assay on Single Cells

**Figure 5:**
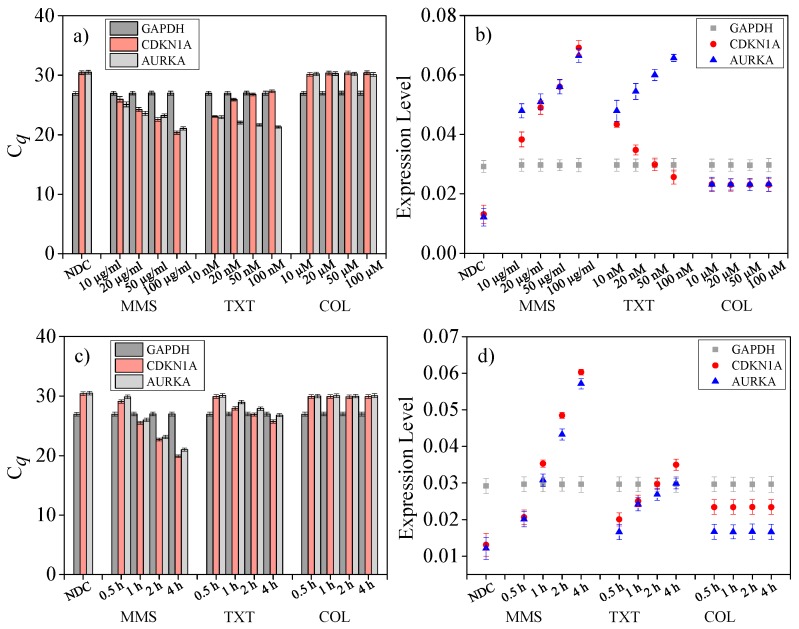
On-chip drug-induced single-cell genotoxicity analysis. (**a**) C*_q_* values of single-cell multiplex RT-qPCR for drug dose analysis; (**b**) Expression levels of *GAPDH*, *CDKN1A*, and *AURKA* genes for drug dose analysis; (**c**) C*_q_* values of single-cell multiplex RT-qPCR for drug treating time analysis; and (**d**) Expression levels of *GAPDH*, *CDKN1A*, and *AURKA* genes for drug treating time analysis. (NDC means ‘no drug treated control’).

Delivering drug reagents with different concentrations to the on-chip immobilized individual cells and incubating for 1 h (37 °C, 5% CO_2_) followed by cell lysis and downstream RT-qPCR, then the drug dose effect on single-cell genotoxicity, was quantified as shown in Figure 5a,b. All of these results were based on five repeated on-chip multiplex RT-qPCR tests.

First, for *GAPDH* from single cells treated by MMS with concentrations ranging from 10 to 100 µg/mL, the C*_q_* values were 26.97 ± 0.32 (Figure 5a). Similar results can be found in TXT (10 to 100 nM) and COL (10 to 100 µM) concentration-related tests. As a housekeeping gene in human, *GAPDH* is expressed at relatively constant levels in most non-pathological situations. These data demonstrated a consistency in *GAPDH* gene expression levels with different chemical stimuli.

Next, for *CDKN1A*, C*_q_* values with MMS concentration increasing from 10 to 100 µg/mL are shown in Figure 5a. MMS can methylate DNA predominantly on N7-deoxyguanosine and N3-deoxyadenosine causing DNA strand damage [44]. While the cells were stimulated by this chemical, as a regulator gene of cell cycle progression at G1 and S phase, *CDKN1A* encodes cyclin-dependent kinase inhibitor protein for binding to and inhibiting the activity of cyclin-CDK2, -CDK1, and -CDK4/6 complexes [45]. This regulation effect in gene expression has been quantified in Figure 5b. Here, the expression levels was defined as E = ΔRn_e_ /C*_q_*, in which ΔRn_e_ meant the endpoint ΔRn value of a 35-cycle qPCR indicating the amplification yield. Expression levels of *CDKN1A* increased monotonously with dosage in MMS tests. However, in TXT tests, expression levels of *CDKN1A* decreased with greater drug concentrations (10 to 100 nM). This phenomenon can be interpreted by the theory that the exposure of MDA-MB-231 cells to TXT induces up-regulation of p53-related genes (including *CDKN1A*) [46,47]. With a higher concentration, the drug effect is more associated with G2/M related transcripts. Finally, treated by COL with different concentrations (10 to 100 µM), the C*_q_* values of *CDKN1A* were close to the data in Figure 4d indicating the stimulus induced alterations in gene expression level of *CDKN1A* was undetectable. As a tubulin targeting compound which inhibits microtubule formation [48], COL-induced DNA damage in a less efficient way than MMS treatment when time length was below 1 h. As the abundance of housekeeping genes are commonly higher than functional genes, the expression levels of *CDKN1A* in this scenario were lower than *GAPDH* (Figure 5b).

Finally, for *AURKA*, C*_q_* values with MMS concentration increased from 10 to 100 µg/mL are shown in Figure 5a. Similar to *CDKN1A*, *AURKA* regulates the cell cycle by encoding the mitotic serine/threonine kinases. Then, MMS-induced DNA damage regulated *AURKA* expression levels, in turn. While exposing to TXT with increased dosage (10 to 100 nM), expression level of this gene was also upregulated and surpassed housekeeping gene expression levels (Figure 5b). The results verified that by induction of both apoptosis and G2/M cell cycle arrest in a dose-dependent manner, TXT can affect *AURKA* expression levels to suppress cell growth [49]. Finally, the 1-hour COL treatment induced DNA damage and related gene expression regulation were not significant. The principle of this phenomenon was similar to the discussion of *CDKN1A* analysis above.

### 4.8. Drug Treat Time Length Assay on Single Cells

Pipetting drug reagents with fixed concentrations (MMS at 10 µg/mL, TXT at 10 nM, and COL at 10 µM) to in vitro cultured MDA-MB-231 cells, and incubating for varied time lengths, followed by on-chip cell trapping, lysis, and downstream RT-qPCR, the effect of treating time length on single-cell genotoxicity was quantified as shown in Figure 5c,d. All of these results were based on five on-chip multiplex RT-qPCR tests.

First, for the housekeeping gene *GAPDH*, with varying time length (0.5 to 4 h), the expression levels of treated single MDA-MB-231 cells were constant (26.97 ± 0.32), demonstrating no significant alteration in *GAPDH* gene expression after MMS, TXT, or COL treatment. Next, for *CDKN1A*, C*_q_* values with MMS treating time were increased from 0.5 to 4 h are shown in Figure 5c. The corresponding expression levels increased with time length growing (Figure 5d). Furthermore, based on these data, it can be concluded that 0.5 h treatment of MMS drug was virtually unable to affect *CDKN1A* expression. After in-culture treating for over 1 h, the expression levels of the functional gene was higher than housekeeping gene, which was consistent with data in Figure 5b. In TXT related tests, *CDKN1A* reached higher expression levels with increased time length. In particular, compared with an on-chip one-hour assay (Section 4.7), the differential value of expression level between *CDKN1A* and *GAPDH* was lower. Since treating dosages and time lengths were identical in this scenario, the data indicated that on-chip single-cell drug treatment caused much more DNA damage than treatment in culture. This phenomenon verified that intercellular interactions in bulk population may affect the response of an individual cell to stimuli which can be refrained by assays at the single-cell level [29]. More microfluidic-based cancer heterogeneity studies at the single-cell level will be investigated at the next stage. Similarly, in COL-related tests, C*_q_* values and expression levels were constant with treating time length variation.

Finally, for *AURKA*, C*_q_* values with MMS treating time increased from 0.5 to 4 h are shown in Figure 5c, and the corresponding expression levels increased with treating time length growing (Figure 5d). This trend can also be found in TXT testing. In the COL test, C*_q_* values and expression levels were stable with varied treating time length.

## 5. Conclusions

Single-cell analysis in microfluidics holds the potential for improving fundamental biomedical research and clinical settings. This work presents a microchip for fully-integrated single-cell gene expression analysis and genotoxicity detection. Treated by methyl methanesulfonate, docetaxel, and colchicine, the dose-dependent and time-dependent gene expression levels of *CDKN1A* and *AURKA* from individual MDA-MB-231 cells have been measured using multiplex RT-qPCR. Throughput was set to be 18, and can be further improved following the same approach. Results from microchip characterization and drug-induced single-cell genotoxicity assays demonstrated the utility of this approach and device for potentially enabling precision single-cell gene expression profiling and cancer research.

## Supplementary Materials

The following are available online at http://www.mdpi.com/1424-8220/16/9/1489/s1. Figure S1: Layout design of the multi-layer microchip. Figure S2: Layout design of the on-chip heater and temperature sensor. Figure S3: (a) 3-D modeling of the microchip in COMSOL; and (b) FEM discretization of the microchip. Figure S4: (a) A microchip prototype; (b) A unit is activated for cell trapping; and (c) Simulation results of the single-cell trapping process. Figure S5: Simulation results of the single-cell trapping process with parametric inlet flow velocity. Figure S6: Fabrication process of the microchip following standard soft photolithography. Figure S7: LabVIEW graphical program and the corresponding front panel for on-chip temperature control of RT-qPCR. Figure S8: Protocol of the on-chip RT-qPCR validation test. Figure S9: End-point fluorescent intensity value of 35-cycle PCR. Figure S10: Corresponding filters and excitation/emission spectra for reporter and reference dyes. Table S1: Properties and classification of the materials.
