# Supplementary material for: A Microchip for Integrated Single-Cell Gene Expression Profiling and Genotoxicity Detection
Source: Sensors (Basel). 2016 Sep 14;16(9):1489. doi: 10.3390/s16091489 (PMC5038763; doi:10.3390/s16091489)
Supplement: Supplementary file 1 [file sensors-16-01489-s001.pdf]

Supplementary Materials: A Microchip for Integrated Single-Cell Gene Expression Profiling and Genotoxicity Detection

Hui Dong and Hao Sun

1. Layout Design of the Multi-Layer Chip

In L-edit software (Tanner Research, Inc., Monrovia, CA, USA), the design of the multi-layer microchip has been finished as shown in Figure S1. The square boundary of the layers defined a 5 × 7.5 cm zone. The width of channels in the flow layer was designed to be 0.5–0.7 mm, while the channel width of control layer was 0.5 mm. Additionally, cross markers (for AZ 4620 and Su-8 mold fabrication of flow layers) were used to align layers during the device packaging process. Layout design of on-chip heater and sensor is shown in Figure S2.

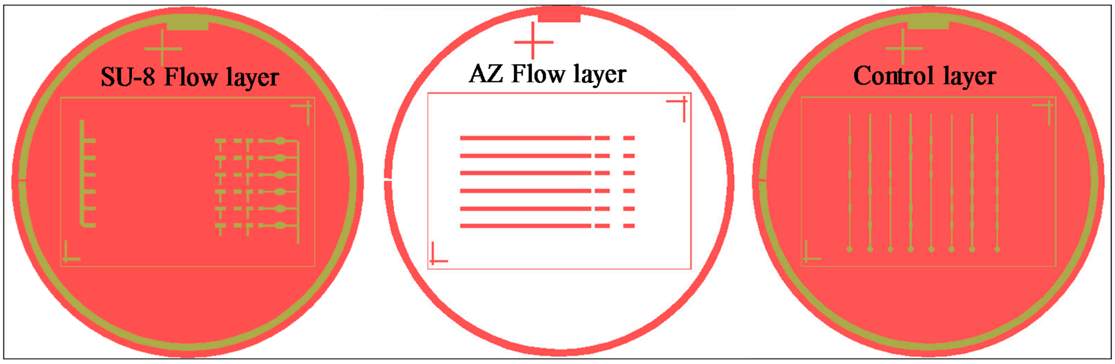

Figure S1. Layout design of the multi-layer microchip.

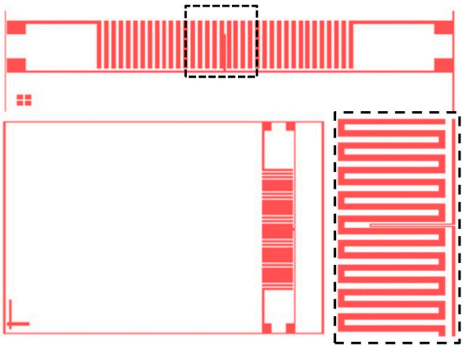

Figure S2. Layout design of the on-chip heater and temperature sensor (Inset shows details of the sensor).

2. Numerical Simulation of On-Chip Single-Cell Trapping

2.1. Theoretical Fundamentals of Fluid-Structure Interaction

For incompressible fluid flow with a length scale of 100 nm to 100 microns, Navier-Stokes equations can be used to describe the flow of fluids:

$$\rho_f \frac{\partial \vec{u}_f}{\partial t} + \rho_f (\vec{u}_f \cdot \nabla)\vec{u}_f = \nabla \cdot [-p_f \vec{I} + \mu_f (\nabla \vec{u}_f + (\nabla \vec{u}_f)^T)] + \vec{F}_f \qquad (1)$$
$$\rho_f \nabla \cdot \vec{u}_f = 0$$

where the subscripts are used to denote the fluid field, ρ and μ are similar to Equation (S1), the fluid density, $u = f(u, v, w)$ is the velocity field (m/s, m/s, m/s), t is the time (s), p is the pressure (Pa), F is the volume force affecting the fluid (N/m^3), μ is the dynamic viscosity of flow (N·s/m^2).

For a cell, when the deformation is within 10% of the sphere diameter (measured by microscope) and the resultant forces are low, linear elastodynamics is used to govern the cell domain:

$$\varepsilon_s = \frac{1}{2}[(\nabla \vec{U}_s)^T + \nabla \vec{U}_s + (\nabla \vec{U}_s)^T (\nabla \vec{U}_s)] \qquad (2)$$

Similarly, the subscripts are used to denote cell domain, ε is the infinitesimal strain tensor and $U = (U, V, W)$ is the solid displacement field (m, m, m). Furthermore, the solid model follows Newton's law of motion and linear elastic stress-strain law:

$$\nabla \cdot \vec{\sigma}_s + \vec{F}_s = \rho_s \frac{\partial^2 \vec{U}_s}{\partial t^2} \qquad (3)$$

$$\vec{\sigma}_s = \vec{D} \varepsilon_s \qquad (4)$$

where σ_s is the Cauchy stress tensor, F_s is the body force per unit volume (N/m^3), D is the stiffness matrix.

Fluid-structure interaction (FSI) combines computational fluid dynamics (CFD) with structural mechanics to investigate the interaction mechanism between fluid flow and cell domain. This FSI coupling appears on the interface of fluid and cell. In particular, the fluid defines the load on the solid surface, while the displacement and velocity of solid is transmitted to fluid. Defining the force on solid surface by fluid to be F_{fs}, and obtain the following equation:

$$\vec{F}_{fs} = -\vec{n} \cdot [-p_f \vec{I} + \mu_f (\nabla \vec{u}_f + (\nabla \vec{u}_f)^T)] \qquad (5)$$

In this study, arbitrary Lagrangian-Eulerian (ALE) method is employed to solve the FSI model, which combines the fluid flow formulated by an Eulerian description and a spatial frame with solid mechanics formulated by a Lagrangian description and a material (reference) frame. Therefore, the velocities of moving mesh (u_m), solid and fluid has the following relationship:

$$\vec{u}_m = \frac{\partial \vec{u}_s}{\partial t}, \quad \vec{u}_f = \vec{u}_m \qquad (1)$$

2.2. Boundary and Initial Conditions

The carrier fluid is driven *via* the pressure difference by a syringe pump. At the inlet boundary, the flow is defined to be laminar with a parabolic velocity profile and velocities of three coordinate directions are set to be constant (3×10^{-5} m/s in –x direction and zero in either the y- or z-axis). At the outlet boundary, the condition is defined as vanishing viscous stress along with a Dirichlet condition on the pressure:

$$p_f = 0, \quad \mu_f (\nabla \vec{u}_f + (\nabla \vec{u}_f)^T)\vec{n} = 0 \qquad (2)$$

At the wall boundary of fluid, no-slip condition is applied, that is:

$$\vec{u}_f = 0 \tag{3}$$

Additionally, the cell surface is selected to be FSI interface boundary and the velocity (initial conditions) of the cell is zero.

2.3. Simulation Set-up

A 3-D geometrical model of the microfluidic system was constructed as shown in Figure S3a, 3a and the properties and classification of the materials were fixed and listed in Table S1.

Table S1. Properties and classification of the materials.

	PDMS	Carrier Flow	Cell	Au	SU-8	SiO_2 Substrate
Density (kg/m^3)	1030	1000	1050	19,300	1219	2400
Young's modulus (GPa)	0.02	/	6.0	79.0	2.0	88.0
Poisson's ratio	0.49	/	0.41	/	/	0.25
Dynamic Viscosity (Pa·s)	/	1.2×10^{-3}	/	/	/	/
Thermal Capacity (J·kg^{-1}·K^{-1})	1500	3200	/	128	1200	750
Thermal Conductivity(W/m^{-1}·K^{-1})	0.18	0.61	/	318	0.3	0.65
Resistivity (Ω/m)	/	/	/	2.4×10^{-8}	/	/

Then, the geometric model was discretized as shown in Figure S3b. The total element number of the meshed chip was 1,382,241 with 4,215,073 degrees of freedom. An implicit method was adopted to solve the transient issue. According to the Courant-Friedrichs-Lewy condition, the time-stepping size was set to 0.001 s while the terminal time was 10 s. The total consumption time in this study was 13,891 s by a dual-core computer with 16 GB of RAM.

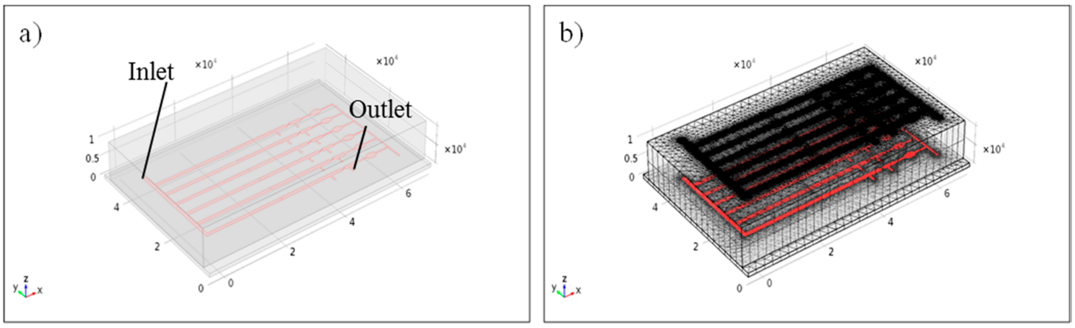

Figure S3. (**a**) 3-D modeling of the microchip in COMSOL; and (**b**) FEM discretization of the microchip.

2.4. Results and Discussion

Corresponding to cell trapping operation illustrated in Figure S4a,b, the von Mises stress distribution on the FSI interface and the slice of velocity magnitude field of carrier flow are obtained and demonstrated in Figure S4c. The stress is a scalar stress value computed from the stress tensor of a solid to determine whether a solid structure will yield subjected to a complex force. The results were found to be symmetrical along a surface created by the PDMS trapping structure outer edge and the center of the cell sphere.

From the above results, we can also conclude that, with an inlet velocity of 3×10^{-5} m/s, the von Mises stress was below 18.6 Pa, which is lower than the stress acting on human vascular endothelial cells. Furthermore, the peak value of velocity magnitude is 7.65×10^{-5} m/s and, therefore, the maximum Reynolds number in this scenario is calculated to be 1.52×10^{-3}. The results indicate that, in the flow field, there are no cross-currents perpendicular to the direction of flow, nor eddies or swirls of fluids.

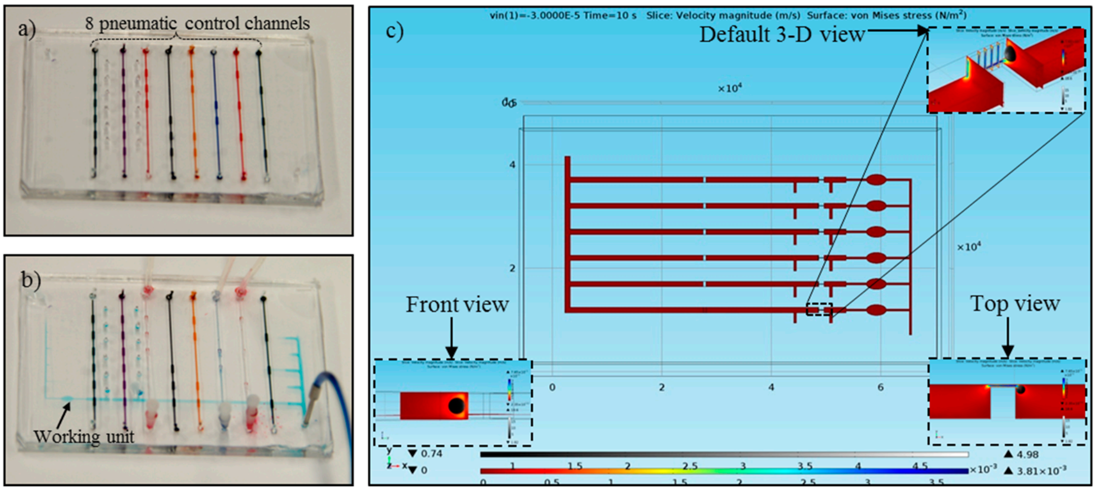

Figure S4. (**a**) A microchip prototype; (**b**) A unit is activated for cell trapping; and (**c**) Simulation results of the single-cell trapping process (for simplification in the FSI study, the on-chip thermal control model, including a temperature sensor and heater, have been omitted).

Furthermore, a parametric solver was used to evaluate the varied flow stress on cell surface with different inlet flow rate (3~30 10^{-5} m/s) conditions. The results (Figure S5) demonstrated that with an inlet velocity below 12 × 10^{-5} m/s, the equivalent von Mises stress was 19.8 dyn/cm^2 which was found to be around the stress acting on normal human vascular endothelial cells. For inlet flow with a higher rate, the stress would impair cell viability.

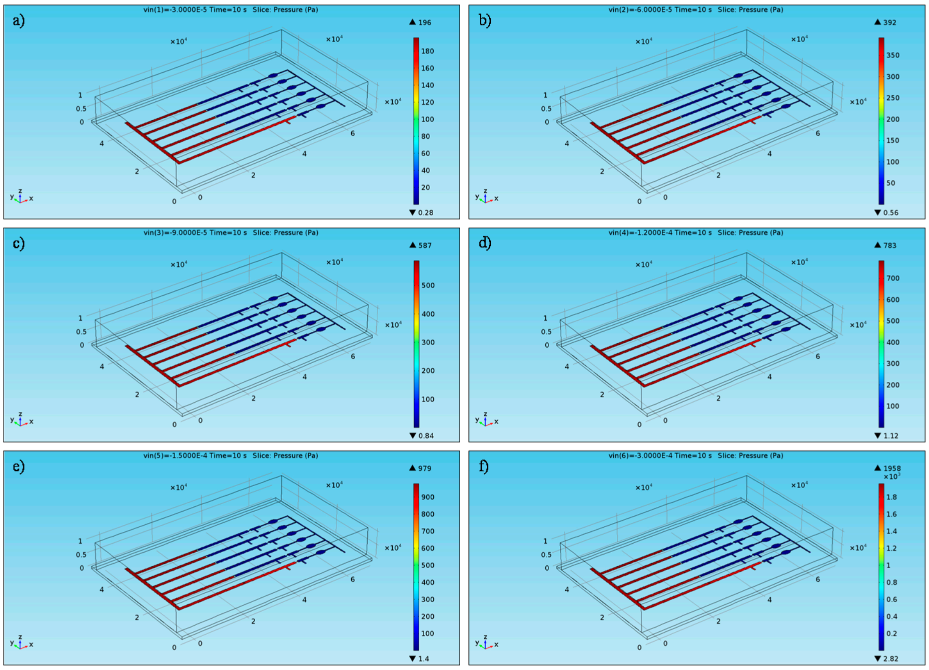

Figure S5. Simulation results of the single-cell trapping process with parametric inlet flow velocity (**a**) Simulation results with the inlet flow velocity of 3 × 10^{-5} m/s; (**b**) Simulation results with the inlet flow velocity of 6 × 10^{-5} m/s; (**c**) Simulation results with the inlet flow velocity of 9 × 10^{-5} m/s; (**d**) Simulation results with the inlet flow velocity of 12 × 10^{-5} m/s; (**e**) Simulation results with the inlet flow velocity of 15 × 10^{-5} m/s; (**f**) Simulation results with the inlet flow velocity of 30 × 10^{-5} m/s.

3. Microchip Fabrication

Chip fabrication was based on the standard soft photolithography as shown in Figure S6

Passivation: The glass slide bearing the heater and sensor was passivated/protected by sequentially spin coating and curing a 10 μm layer of SU-8 photoresist (4000 rpm for 45 s, 95 °C for 10 min for curing the photoresist), followed by a 10 μm of PDMS (5000 rpm for 1 min, 80 °C for 20 min for curing PDMS) for device packaging. Oxygen plasma was employed to bind the passivation layer with the PDMS microfluidic device. After each RT-qPCR use, the PDMS microfluidic device was peeled off while the heater and sensor can be reused.

SU-8 photolithography for the mold: The SU-8 mold features were 15 μm high for the flow layer and 80 μm high for the control layer. The protocol for the 15 μm high flow layer was adapted from the manufacturer guidelines (Gersteltec Sarl Inc., Pully, Switzerland). First, at room temperature, permanent epoxy negative photoresist SU-8 was spin-coated on a cleaned 4 inch silicon substrate at a speed of 3200 rpm for 45-60 s with an acceleration of 300 rpm/s. Then, the coated SU-8 photoresist was placed on a level hotplate for 10~15 min at 95 °C. Next, the baked photoresist was exposed under UV light (filtered to a 365 nm wavelength) at a dose of 130~150 mJ/cm^2 using a mask aligner (URE-2000A/55 Mask Aligner, Chengdu, China). After exposure, the patterned SU-8 was placed on a hotplate for 4 min at 95 °C. Then, the exposed SU-8 photoresist was sprayed with SU-8 developer for 2~3 min. At the end of the development, the exposed photoresist was sprayed and washed with fresh SU-8 developer for approximately 10 s, followed by a second spray/wash with isopropyl alcohol (IPA) for another 10 s. The mold was dried with pressurized nitrogen gas. Finally, the silicon substrate bearing the SU-8 features was baked (150–250 °C) for 30 min to ensure that SU-8 properties do not change with thermal cycling. Similarly, according to the manufacturer's guidelines (Gersteltec Sarl Inc., cPully, Switzerland), we built an 80 μm high mold which had been confirmed by a non-contact 3D measurement system (Micromesure 2 profilometer, STIL, Ontario, NY, USA).

Evaporation resist film: From the manufacturer (Thermo Scientific), the vapor barrier (optical adhesive film) is composed of polypropylene and was designed for creating a secure seal across a microplate to prevent evaporation. The thickness of the film was 0.1 mm as measured by a micrometer gauge. A two-step PDMS casting process was employed to embed the vapor barrier above the reaction chamber. Initially, the base and curing reagent of PDMS were mixed in a 10:1 ratio. The mixture was degassed for 45 min and then was spin-coated on the mold at a speed of 4000 rpm for 45 s with an acceleration of 300 rpm/s followed by baking at 72 °C for 15 min. Next, a piece of adhesive film was stamped on PDMS at the region of the reaction chamber. After that, 15 mL uncured PDMS (10:1) was poured on the solid-state PDMS layer and baked to finalize the barrier embedding (See Figure S6).

Device package: The mechanism of PDMS bonding is related to the breaking of bonds on each surface of PDMS during treatment followed by the formation of Si-O-Si bonds when the two surfaces of PDMS are brought into contact. The PDMS layers or substrates were first installed in the process chamber of the DQX-2s RIE system (Ketan Inc., Zhengzhou, China). The samples were treated with oxygen plasma at 250 mTorr pressure by a power of 50 Watt for 4 s.

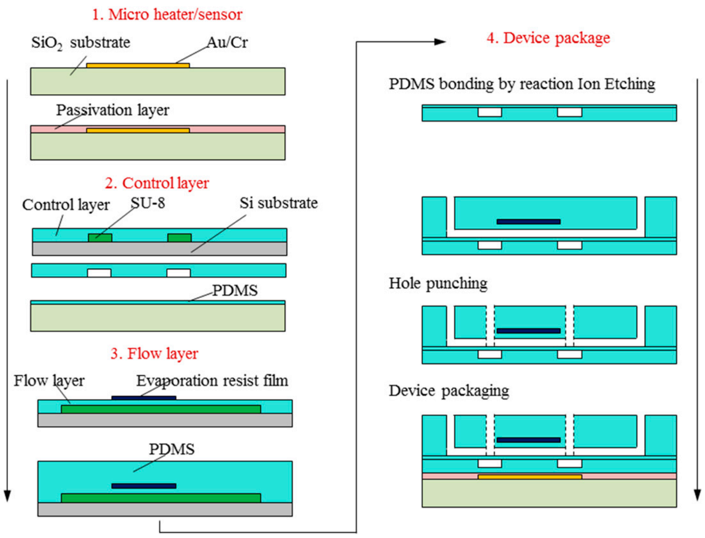

Figure S6. Fabrication process of the microchip following standard soft photolithography.

4. On-chip Temperature Control Program

A LabVIEW (National Instruments Corp., Austin, TX, USA) graphical program as shown in Figure S7 was used for on-chip temperature control.

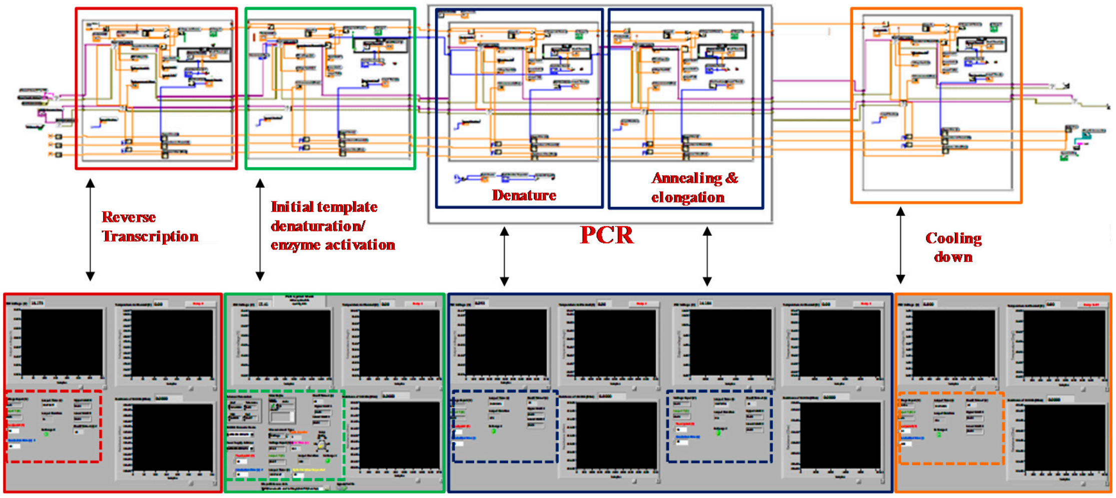

Figure S7. LabVIEW graphical program and the corresponding front panel for on-chip temperature control of RT-qPCR. The temperature of an integrated RT-qPCR is controlled by the software using a proportional integrated derivative method. Resistance readings are collected by the sensor on the chip, send through the General-Purpose Interface Bus(GPIB) card to the computer to calculate the temperature. The data for the temperature adjustments are then sent through the GPIB card back to the chip where the data will drive the heater.

5. On-chip RT-qPCR Validation Protocol

First, pipette 0.2 μL XenoRNA to each unit of chip which have been incubated by 0.5 μl bead solution, and mix them for 10 min. Then, pipette the RT master mix (Figure S8) to the chip and flush through the reaction chamber while immobilize bead/RNA by a magnet. After the RT process, until the chip cools down to room temperature, pipette PCR reagents to the chip following the volume in Figure S8. Finally, activate the LabVIEW program for the on-chip 35-cycle PCR. The end-point result of ΔRn is obtained as shown in Figure S9.

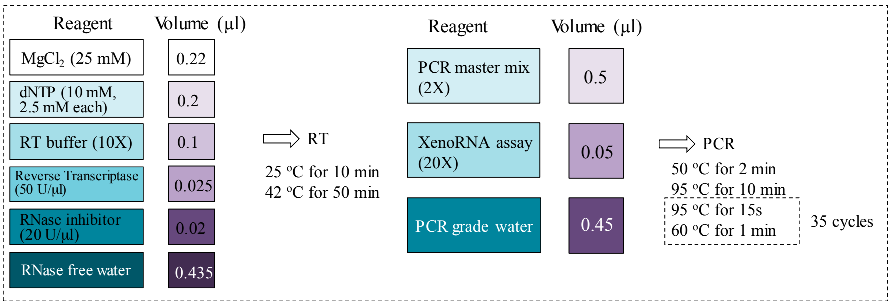

Figure S8. Protocol of the on-chip RT-qPCR validation test.

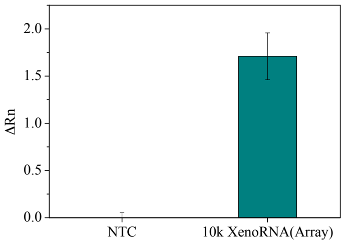

Figure S9. End-point fluorescent intensity value of 35-cycle PCR.

6. On-chip Single Cell Gene Expression Profiling Procedures

The gene expressions of untreated single cells were assayed for the induction of GAPDH, CDKN1A, and AURKA using target-specific hydrolysis probes/primers. HEX, FAM, and CY3 reporters were used to follow each individual amplification reaction which were compatible with the excitation and emission filters of the microscope system. Correspondingly, Black Hole Quencher-1 (BHQ-1), BHQ-2, and BHQ-1 were employed for fluorescence quenching. Filters of HPF 1295, HPF 1265, and HPF 1310 were selected for detecting HEX, FAM, and CY3 dyes. HPF 1340 was used to detecte ROX. The wavelengths of excitation and emission filter are illustrated in Figure S10 (Newport Corporation, Franklin, MA, USA).

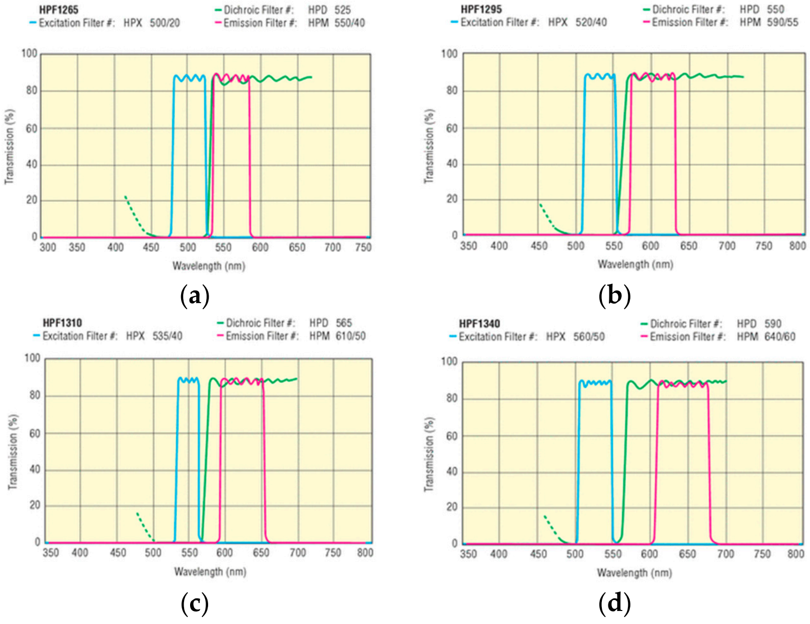

Figure S10. Corresponding filters and excitation/emission spectra for reporter and reference dyes. (**a**) Excitation/emission spectra of the HPF 1265 filter; (**b**) Excitation/emission spectra of the HPF 1295 filter; (**c**) Excitation/emission spectra of the HPF 1310 filter; (**d**) Excitation/emission spectra of the HPF 1340 filter. (Copyright by Newport Corp.).
